# Increased Expression of TRPV1 in the Central Nucleus of the Amygdala Is Involved in Orthodontic Pain in Rats

**DOI:** 10.3390/ijms262311296

**Published:** 2025-11-22

**Authors:** Rui Wang, Weining Wang, Yuxin Kang, Yuhuan Jiang, Xiaoyu Tang, Yaxing Shu, Jiayi Zhou, Zhiping Hu, Shuang Wang, Hu Qiao

**Affiliations:** Key Laboratory of Shaanxi Province for Craniofacial Precision Medicine Research, College of Stomatology, Xi’an Jiaotong University, Xi’an 710004, China

**Keywords:** tooth movement pain, CeA, TRPV1, behavior assessment, central regulation

## Abstract

Transient receptor potential vanilloid type 1 (TRPV1) is known to gate nociceptive signals, while the central amygdala (CeA) plays a key role in encoding pain and avoidance behaviors; however, whether TRPV1 within CeA mediate orthodontic tooth-moving pain remains unclear. To investigate this, we examined the role of TRPV1 in the CeA using a rat model of experimental tooth movement. A total of 118 Sprague-Dawley rats were divided into control, 30 g, 50 g, and 80 g force groups. Micro-CT confirmed successful tooth movement, and the 50 g force was selected as optimal. Pain was assessed by mechanical hypersensitivity using the von Frey test, face-grooming, and the Rat Grimace Scale (RGS). All measures showed maximal changes at day 1 and returned to baseline by day 7. Immunohistochemistry and western blot analysis in the 50 g group revealed upregulation of TRPV1 in the CeA following force application, a trajectory that paralleled the pain behavioral responses. These findings indicate that experimental tooth movement upregulates TRPV1 in the CeA and that this channel contributes to orthodontic pain.

## 1. Introduction

Pain is defined as an unpleasant feeling and emotional experience accompanied by actual or potential tissue damage. The prevalence of malocclusion in Chinese children/adolescents increased year by year, from 40% in the early 1960s to 67.82% in 2000 [1]. Most orthodontic patients experience pain during the treatment process, which can lead to major dissatisfaction and discontinuation of long-term treatment [2]. Furthermore, emerging evidence indicates that orthodontic pain extends beyond sensory discomfort, impairing quality of life via compromised mastication, speech articulation, and transient cognitive deficit [3]. Thus, exploring mechanisms to alleviate orthodontic pain has become increasingly imperative.

Orthodontic pain is thought to be caused by the stimulation of periodontal and pulp tissues, which can be activated by orthodontic force and local inflammatory mediators. These impulses are transmitted via Aδ and C fibers to the trigeminal ganglion before projecting centrally [4]. The amygdala, as a component of the limbic system [5], is closely related to the generation and regulation of pain [6], as well as anxiety [7]. Studies show that electrical stimulation of the maxillary anterior teeth induces pain and activates the amygdala, suggesting its important role in encoding oral-facial pain [8]. The amygdala mainly consists of the basal lateral amygdala, central amygdala (CeA), and medial amygdala; among them, the CeA integrates multiple sensory signals including nociceptive sensory information [9].

The CeA contains diverse nociceptive neurons and serves as a pivotal hub for encoding and processing nociceptive information. It integrates nociceptive inputs and, through its extensive downstream projections, modulates activity in the hypothalamus, substantia innominata, and brainstem regions, thereby playing a critical role in pain transmission and modulation. Research showed that in establishing a rat orthodontic tooth movement model, enhanced c-Fos immunoreactivity was observed in the CeA, and the expression intensity was positively correlated with the force application intensity [10]. Furthermore, supporting evidence shows that after electrolytic lesions were made to bilateral CeA in rats, tooth movement pain behavior was significantly alleviated, indicating that an intact CeA is crucial for the formation of tooth movement pain response [11]. However, the specific mechanisms remain unclear, and thus further exploration is required.

Transient receptor potential vanilloid type 1 (TRPV1) is a polymodal nociceptive transducer that is expressed widely in the cortex, hippocampus, CeA, dentate gyrus, hypothalamus, striatum, thalamus, nucleus of the trigeminal nerve, and inferior olive [12]. Studies have shown that individuals with TRPV1 mutations exhibit no sensitivity to topical capsaicin application in the oral cavity or on the skin, and display no behavioral aversion to capsaicin exposure. These findings provide direct evidence of TRPV1-mediated pain-related functional alterations in humans [13]. Meanwhile, TRPV1 was significantly increased in the trigeminal nerve 1 d after rat tooth movement model establishment, indicating that TRPV1 was mainly activated during early responses [14]. However, few studies have focused on the roles of TRPV1 in the central nervous system. Accordingly, we sought to determine the role of TRPV1 in the CeA in regulating tooth movement-induced pain.

Based on our previous findings [11], this study aimed to investigate pain behavior and TRPV1 expression in the CeA during tooth movement using behavioral and molecular biology techniques in order to elucidate the underlying neural mechanisms and provide a theoretical basis for potential interventions for orthodontic pain.

## 2. Results

### 2.1. Micro-CT and Rate of Tooth Movement

**Figure 1:**
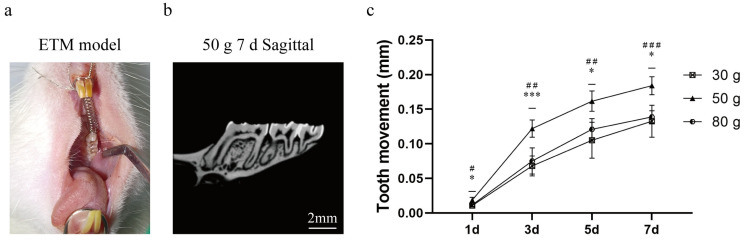
Micro-CT performance of ETM. (**a**) ETM model diagram. (**b**) ETM image of 50 g 7 d. (**c**) ETM distance in each group from 1 day to 7 d (mm). Abbreviations: ETM, experimental tooth movement. *n* = 5, * *p* < 0.05, *** *p* < 0.001, 30 g vs. 50 g Group; # *p* < 0.05, ## *p* < 0.01, ### *p* < 0.001, 50 g vs. 80 g Group.

Tooth movement is presented in Figure 1. Tooth movement can be observed based on different magnitudes of force, and the movement distance increases with an increase in force. After established the ETM model, the tooth movement rate of 50 g group was the most ideal group. We speculated that the force of 30 g was small, resulting in limited effects. In contrast, the force of 80 g was too large to move the tooth properly. Accordingly, 50 g was selected as the force magnitude for subsequent experiments.

### 2.2. Face-Grooming Behavior After ETM with 50 g Force

**Figure 2:**
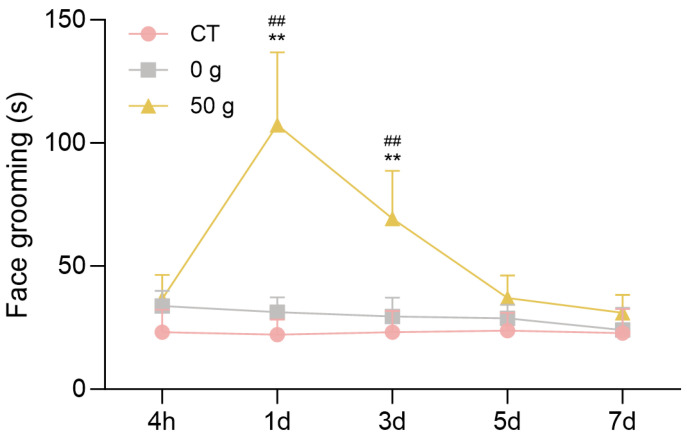
Face-grooming behavior following ETM. *n* = 6, ** *p* < 0.01, 50 g vs. CT Group; ## *p* < 0.01, 50 g vs. 0 g Group.

Changes in the face-grooming activity of rats in each group were observed at 4 h, 1 d, 3 d, 5 d, and 7 d (Figure 2). Under force, the mouth face grooming appeared obviously, and the time duration of face grooming in the 50 g group was significantly higher than those in the CT and 0 g group at 1 d (*p* = 0.0016, CT group vs. 50 g group; *p* = 0.0028, 0 g group vs. 50 g group) and 3 d (*p* = 0.0027, CT group vs. 50 g group; *p* = 0.0064, 0 g group vs. 50 g group). The analysis revealed that 50 g induced obvious pain behavior, and the pain peaked at day 1.

### 2.3. RGS After ETM with 50 g Force

**Figure 3:**
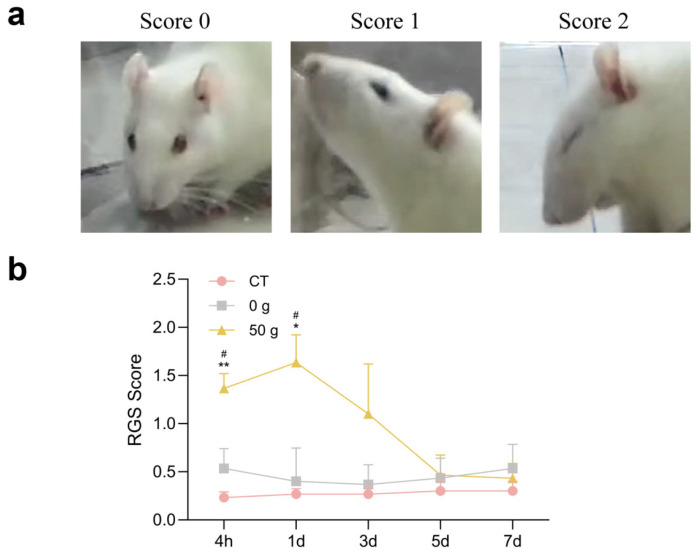
RGS score following ETM. (**a**) RGS coding magnitude. (**b**) Quantitative analysis. *n* = 6, * *p* < 0.05, ** *p* < 0.01, 50 g vs. CT Group; # *p* < 0.05, 50 g vs. 0 g Group.

Changes in the RGS score following initiation of ETM are presented in Figure 3. Under force, the facial expression showed obvious harmful changes, and RGS scores in the 50 g group were significantly higher than those in the CT and 0 g groups at 4 h and 1 d (Figure 3). The results exhibited a similar tendency to that of face-grooming behavior.

### 2.4. Von Frey Filament Test After ETM with 50 g Force

**Figure 4:**
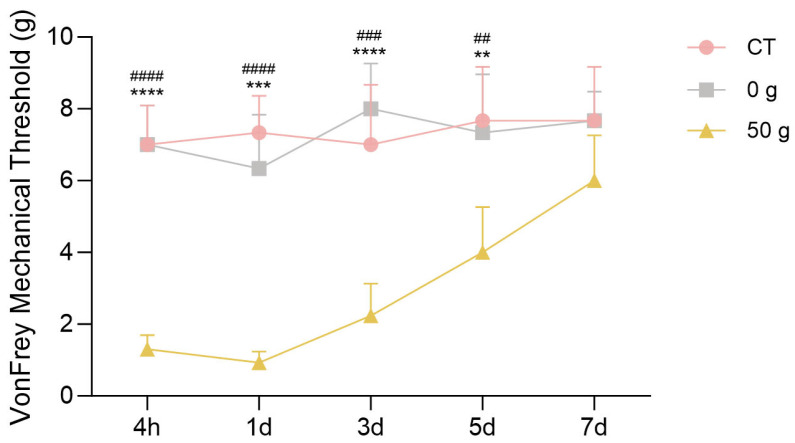
Von Frey filament test after ETM. *n* = 6, ** *p* < 0.01, *** *p* < 0.001, **** *p* < 0.0001, 50 g vs. CT Group; ## *p* < 0.05, ### *p* < 0.001, #### *p* < 0.0001, 50 g vs. 0 g Group.

The results of the von Frey test following the establishment of the tooth movement model are shown in Figure 4. Compared with the control and 0 g groups, the mechanical pain threshold in the 50 g group decreased significantly at the 4 h and 1 day time points, reaching its lowest level at 1 day. Subsequently, a trend of progressive recovery was observed, with the threshold returning to baseline by day 7. In contrast, the control and 0 g groups exhibited stable pain thresholds throughout the experiment.

### 2.5. Expression of TRPV1 in the CeA After ETM

**Figure 5:**
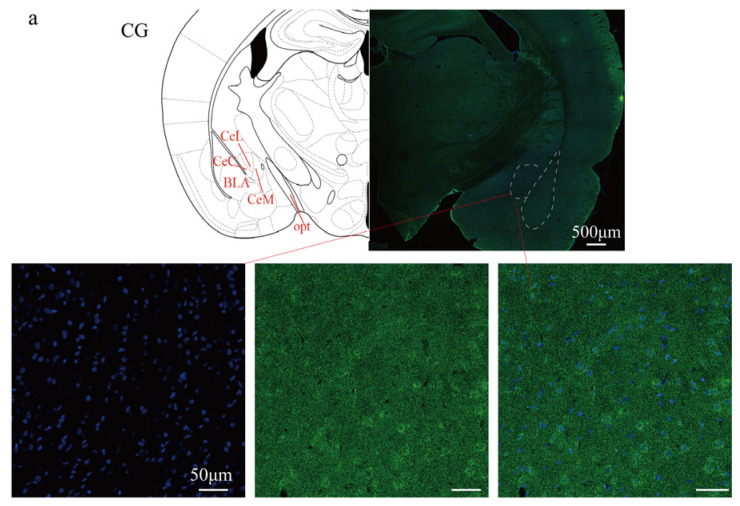

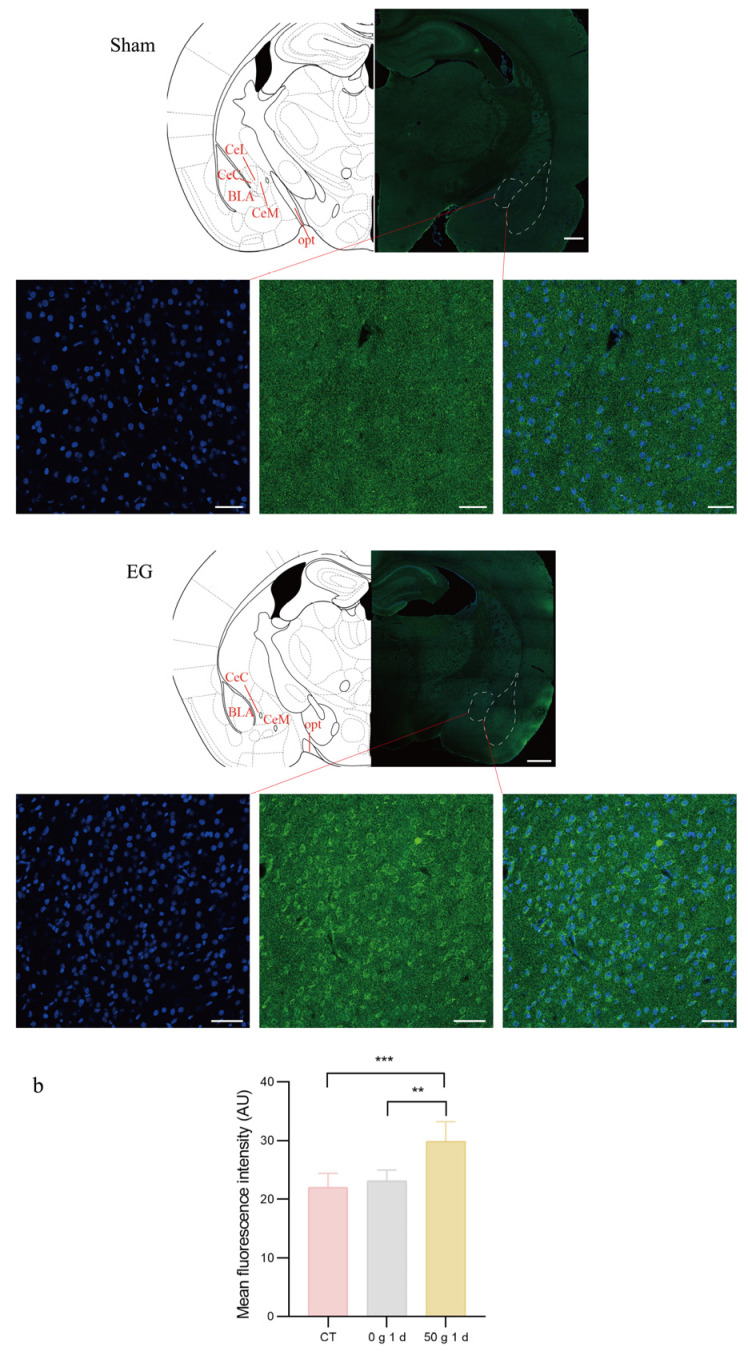
Immunofluorescent expression of TRPV1 in the CeA. (**a**) Immunofluorescent staining diagram. (**b**) Statistical analysis of fluorescence intensity. Cell nuclei were stained with DAPI (blue). The expression of the TRPV1 protein is shown in green. *n* = 6, ** *p* < 0.01, *** *p* < 0.001. Scale bars: 500 μm (overview); 50 μm (magnified views). CeL: Central nucleus of the amygdala, lateral division. CeM: Central nucleus of the amygdala, medial division. CeC: Central nucleus of the amygdala, capsular division. BLA: Basolateral nucleus of the amygdala. opt: Optic tract.

**Figure 6:**
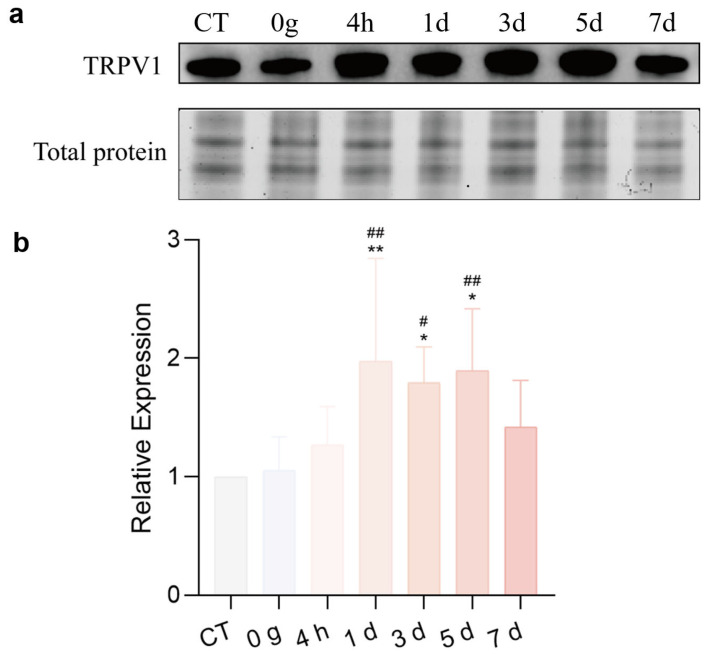
Western blots of TRPV1 in the CeA. (**a**) TRPV1 expressed in the CeA. (**b**) Quantitative analysis. *n* = 6, * *p* < 0.05, ** *p* < 0.01, 50 g vs. CT Group; # *p* < 0.05, ## *p* < 0.01, 50 g vs. 0 g Group.

**Figure 7:**
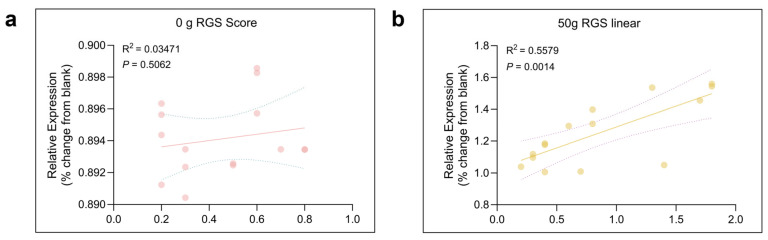
Correlation between the time spent on RGS and expression of TRPV1 in the CeA. Pearson correlation revealed significant correlation between the intensity of TRPV1 and the RGS score in 0 g (**a**) (R^2^ = 0.03471, *p* > 0.05) and 50 g (**b**) (R^2^ = 0.5579, *p* = 0.0014) groups. The solid line summarized this trend, with the dispersion of the individual data points around the line and the dashed line indicating the strength of the relationship.

To investigate the expression of TRPV1 in the CeA after ETM, immunofluorescence analysis was performed. As shown in Figure 5, TRPV1 immunofluorescence (indicated in green) was observed in the CeA. Compared to total protein, TRPV1 expression in the CeA was significantly differentially expressed. TRPV1 was strongly expressed at 1 d and 3 d, as shown in Figure 6. A positive correlation was observed between RGS score and TRPV1 expression in the CeA. Results for the sham-treated (R^2^ = 0.03471, *p* > 0.05) and experimental (R^2^ = 0.5579, *p* = 0.0014) groups are presented in Figure 7.

## 3. Discussion

Pain is a common adverse reaction in the process of orthodontic treatment. Approximately 95% of patients will experience discomfort and pain during orthodontic treatment, which may result in anxiety and negatively impact their mental state. Indeed, some patients may even discontinue follow-up orthodontic treatment [15]. Orthodontic patients generally spend approximately 2 years on the treatment, and there is a high demand for patient compliance during treatment. As pain is a key factor affecting patients’ comfort, it is critical for patients to maintain good compliance during the course of treatment.

Oral and maxillofacial pain is commonly assessed through methods such as face-grooming activity and RGS coding and the von Frey filament test, and von Frey filament test provides the direct measurement of mechanical pain thresholds [16]. The face-grooming behavior includes different actions, such as mouth wiping, forelimb flailing, ear grasping, face-washing strokes, headshakes, and so on. Mouth wiping is a direct and robust behavioral response for pain evaluation after ETM [17]. RGS coding predominantly analyses changes in facial expressions, including orbital tightening, nose/cheek flattening, ear changes, and whisker changes [18]. The von Frey test utilizes filaments to determine the mechanical force eliciting a defensive withdrawal response, providing an objective and quantitative measure of mechanical sensitivity. Thus, we employed a combination of the three tests to assess pain behavior during ETM, and found that pain occurred from 4 h, peaked at 1 d, decreased from 3 d, and finally returned to baseline at 7 d. These results showed the non-evoked pain behaviors (face grooming and RGS) and the evoked mechanical hypersensitivity (von Frey) indicated a similar pain tendency, also verifying that these approaches all remained reliable for evaluating pain responses during orthodontic tooth movement. These findings are consistent with previous research [11,19] and clinical observations of orthodontic pain [20], which also implied that pain occurrence derived from ETM.

In this study, the rate of tooth movement and pain were measured. Orthodontic tooth movement models using SD rats are generally established by placing a stainless steel tension spring between the incisor and molars, which relies on the force of the tension spring to produce tooth movement. Under the continuous action of a 50 g spring force, the first molars of rats could produce the effect of tooth movement on day 3, and the distance of tooth movement increased continuously with the passage of time. From day 1 to day 3, tooth movement was faster in the 30 g and 50 g groups than in the 80 g group, which could be due to excessive stress, osteocyte degeneration, and necrosis in the 80 g group. After 7 days, tooth movement rate was significantly increased in the 30 g and 50 g groups, which may be due to the self-repair of periodontal tissue and active osteoclast phenomenon. In contrast, the basic stagnation of tooth movement in the 80 g group may be due to severe periodontal damage and necrotic tissue removal [21].

The amygdala has been extensively studied in fear conditioning and affective processes; a growing body of research has confirmed its significant involvement in processing nociceptive information and modulating pain [22]. Arimura et al. reported that in a formalin-induced inflammatory pain model, the central amygdala (CeA) is activated, and its chemogenetic inhibition suppresses pain-evoked activations across widespread brain regions, indicating the necessity of CeA in this process [23]. Beyond this, in a rat model of visceral pain induced by colorectal distension, neuronal activity in the CeA was significantly enhanced, as indicated by a marked increase in c-Fos expression [24]. Under various pain models, CeA neurons exhibit increased excitability and are considered crucial for pain-related behaviors. Our previous studies have further confirmed that the integrity of the CeA is essential in orthodontic pain induced by tooth movement, although the underlying mechanisms remain unclear [11]. Moreover, while the CeA’s role in pain is increasingly recognized, its specific functions in orofacial pain—particularly in the context of orthodontic pain—are still poorly understood and require further investigation.

The central nervous system (CNS) neurons responsible for processing nociceptive information are equipped with a diverse array of receptors and ion channels, enabling the precise response to complex physiological and pathological stimuli [25]. Among these, the transient receptor potential vanilloid type 1 (TRPV1) channel has emerged as a pivotal molecular player. TRPV1 contributes to pain sensitization and neuropathic pain development by interacting with inflammatory mediators and modulating the excitability of nerve fibers and neurons [26]. At present, most studies about the effect on TRPV1 regulating orthodontic pain focus on the peripheral nervous system, such as Thammanichanon showed that TRPV1 in the trigeminal ganglion increased significantly on 1 d with tooth movement in rats [27]. However, there are few studies which focus on the regulation of TRPV1 in the central nervous system about orthodontic pain.

In this study, TRPV1 expression in the CeA of rats under orthodontic tooth movement was analyzed. The results highlighted regularity in TRPV1 expression in the CeA among groups. Moreover, TRPV1 expression was positively correlated with RGS coding. These findings suggest that TRPV1 in the CeA may be involved in the exacerbation of orthodontic pain. The absence of a significant correlation between face-grooming and TRPV1 expression in this study contrasts with our previous findings [28]. This discrepancy may be primarily explained by the fundamental difference in TRPV1’s role: its high density in peripheral trigeminal pathways likely dominated the behavioral response in earlier work, whereas its sparse, modulatory expression within the CeA yields a weaker behavioral correlate. Furthermore, the face-grooming assay itself, as a single-metric test, captures a narrower range of behaviors than the composite RGS score, which likely contributed to the lack of correlation here. This highlights the variable sensitivity of behavioral tests and the limitation of relying on any single readout.

Moreover, this study revealed that pain behavior and TRPV1 expression in the CeA peaked at 1 d after establishing the ETM model, and there was a correlation between TRPV1 expression and pain behavior induced by ETM. Notably, TRPV1 was significantly upregulated in the 50 g group compared to the control group. Furthermore, a moderate upregulation was also observed in the 0 g group. We suggest that this mild increase in the 0 g group likely results from the physical presence of the orthodontic appliance itself, whereas active force application (50 g) induced a stronger and more functionally relevant expression upregulation. The present study provides direct evidence of TRPV1 upregulation within the central nervous system, with a specific focus on the CeA. While our previous research established TRPV1 elevation in the trigeminal ganglion [28], the functional connectivity between these peripheral and central pathways remains to be experimentally verified. Consequently, we cannot yet determine whether the observed central TRPV1 expression results from local synthesis or peripheral mediation. Elucidating this precise mechanism represents an important objective for future studies. Although TRPV1 serves as a key pain mediator in inflammatory processes, our findings are derived from a short-term rat model. Confirming its potential as a therapeutic target will require validation in prolonged models and more comprehensive experimental approaches.

Meanwhile, anxiety is common in orthodontic patients, and there is an interaction between orthodontic pain and negative emotions [29], and CeA may play an important role in this process [11]. After processing the pain signals, CeA projects the nociceptive information to the cortex then produces anxiety. At the same time, the cortex also transmits information about anxiety to the amygdala, which can ultimately influence information processing in the trigeminal nucleus and ultimately regulate pain [30]. Thus, the subsequent studies will explore the role of CeA in regulating anxiety associated with orthodontic treatments. Finally, the production of pain and emotion is the result of the interaction of various nuclei in the central nervous system, so our subsequent research is going to explore the nuclei interacting with CeA, to find a neural pathway related to orthodontic pain regulation.

In summary, this study demonstrates that orthodontic pain correlates with increased TRPV1 expression in the central amygdala, with behavioral and molecular changes exhibiting a corresponding pattern. These findings suggest the potential involvement of TRPV1 within CeA in pain processing and establish a foundation for further investigation into its functional role in orthodontic tooth movement pain.

## 4. Materials and Methods

### 4.1. Experimental Design and Animals

The experiments were conducted using 8-week-old male Sprague-Dawley rats weighing between 200 and 250 g were obtained from the Experimental Animal Centre of Xi’an Jiaotong University Health Science Centre (Xi’an, China). Rats were nursed in standard cages with food and water on a 12 h light/12 h dark cycle in a temperature-controlled (21 ± 1.5 °C) room. Rats were allowed to acclimate to the housing conditions for at least 5 days before commencement of experiments. The applied force was precisely controlled by adjusting the extension of the precision spring. Based on preliminary experiments that identified 50 g as the optimal force, rats were allocated into three experimental groups: blank control (CT), sham (0 g), and 50 g groups. Each group was examined at 0 h, 4 h, 1 d, 3 d, 5 d, and 7 d after the force. Face-grooming behavior and the Rat Grimace Scale (RGS) were used to observe behavioral responses to nociceptive stimulation. Tooth movement distance was evaluated using micro-CT. TRPV1 expression in the CeA was investigated using immunofluorescence and western blotting. Every effort was made to minimize animal discomfort, in accordance with the ethical guidelines for animal research [31]. Experimental procedures were conducted in accordance with the National Institutes of Health Guide for the Care and Use of Laboratory Animals, and were approved by the Biomedical Ethics Committee of Xi’an Jiaotong University Health Science Center (Approval NO. 2020-386).

### 4.2. Experimental Tooth Movement (ETM)

**Figure 8:**
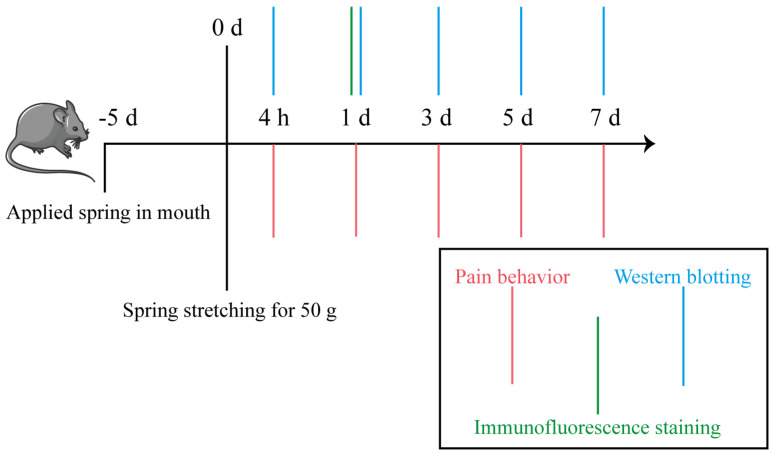
Flow chart of behavioral and molecular biology experiments.

Rats were anesthetized with Avertin (i.p.; 10 mL/kg). A fixed stainless steel spring closed-coil custom-made spring appliance (SUS304-WPB, Guangzhou, China) was constructed to perform mesial movement of the right maxillary first molar [32]. Specifically, the closed-coil spring hooked the right maxillary first molar and upper incisors, then a force gauge was used to determine the force value of the spring after stretching. A shallow groove was made on the neck of the upper incisors, and glass ionomer was used as a cover to enhance retention. After established the ETM model, the behavioral testing and molecular biology analysis process were conducted at different time points, as shown in Figure 8.

### 4.3. Face-Grooming Behavior

Rats were monitored for directed face-grooming behavior in transparent plastic cages (40 cm × 40 cm × 40 cm) placed in a room with 45 dB background noise between 9 AM and 12 AM. Rats were allowed to acclimate to the housing conditions for at least 15 min before recordings were performed. Behavior was recorded by cameras for a period of 10 min at each time point. The positive action was raising hands to wipe mouth corner, and recorded the duration time of the face-grooming behavior. The test session was subsequently analyzed by two independent observers who were blinded to the treatment. The average of three estimations was used to yield a mean value for each animal.

### 4.4. Rat Grimace Scale (RGS)

When animals receive nociceptive stimuli, facial expressions can be an obvious change, such as orbital tightening, flattening of the nose/cheeks, ear changes, whiskers changes and so on, which can be scored using the grimace scale. In the evaluation of the grimace scale, cameras were placed on both sides of a transparent box, the behavior of animals within 30 min was recorded, and clear images of the rat’s face were captured every 3 min; score “0” represents no pain, score “2” represents the most pain, and score “1” represents moderate pain. In total, 10 photos were captured and encoded for each rat. RGS scoring was performed by two experimenters in a blinded manner [16].

### 4.5. Von Frey Filament Test

Rats were acclimated to the mesh-testing apparatus prior to ensure a quiet, unrestrained state. Mechanical withdrawal thresholds were determined by applying von Frey filaments (Yuyan, Shanghai, China) to the surgical-side whisker pad in ascending order of force. A positive response—defined as withdrawal, aversive movement, or asymmetric facial grooming—was recorded if three of five stimulations at a given force elicited a reaction. The threshold was confirmed when the next lower filament in the series failed to evoke a withdrawal response. Behavioral testing was conducted at 4 h and 1, 3, 5, and 7 days post-surgery.

### 4.6. Micro-CT

Tooth movement distance was measured using micro-CT (PerkinElmer, Waltham, USA). The rat’s head was placed into a position that was symmetrically aligned to the three spatial axes within a recordable cylindrical volume of 4 cm × 4 cm × 4 cm. The distance of the most prominent point from the distal side of the first molar to the mesial side of the second molar of the maxillary was measured and analyzed.

### 4.7. Immunohistochemistry

The distribution of TRPV1 receptors in the CeA was analyzed using immunofluorescence and western blot after ETM in the control and 50 g force experimental rats (*n* = 6 rats per group). Rats were killed with an overdose of 10% chloral hydrate and perfused with 150 mL of saline solution followed by 500 mL of 4% paraformaldehyde. Brains were removed and postfixed for 12–24 h in 4% paraformaldehyde in 0.1 M phosphate buffer (PB). The brains were then placed in a 30% sucrose solution for another 72 h. Following sucrose cryoprotection, 25 μm sections were cut on a freezing sliding microtome. Sections were stored at 4 °C until the tissue was prepared for immunofluorescence [33].

After three washes in phosphate-buffered saline (PBS), tissue sections were incubated for 15 min at room temperature (23 ± 3 °C) in a blocking solution of QuickblockTM Blocking Buffer for Immunol Staining (Beyotime Biotechnology, China). Slides were incubated with rabbit anti-TRPV1 IgG (1:500 dilution; Alomone, Israel) at 4 °C for 48 h. The samples were then incubated with a 488-conjugated goat anti-rabbit IgG (H + L) secondary antibody (1:500 dilution; Immunoway, American) for 4–6 h at room temperature. Sections were examined under a fluorescence microscope (Olympus, Tokyo, Japan) equipped with a digital camera.

### 4.8. Western Blot Analysis

Protein extraction of CeA tissue was conducted with RIPA lysis buffer and a protease inhibitor cocktail (Roche, Penzberg, Germany). BCA (bicinchoninic acid) method (Beyotime Biotechnology, Shanghai, China) was used to determine the protein concentration. A total of 30 μg of protein of each sample was separated in 10% TGX Stain-Free polyacrylamide gels (Bio-Rad, Hercules, CA, USA). Total protein data were obtained using a GelDoc Go Imaging System Ordering (Bio-Rad, Hercules, CA, USA) and transferred onto a membrane. After blocking with 5% skim milk powder at 25 °C for 1 h, the following primary and secondary antibodies were used: polyclonal rabbit anti-TRPV1 (Santa Cruz, Santa Cruz, CA, USA), goat-anti-rabbit IgG (1:5000 dilution; CST, New York, NY, USA). The densities of the protein blots were analyzed using Software Imagelab 6.1 (Bio rad, Hercules, CA, USA). Total protein was employed as an internal control to normalize target protein levels, a method widely validated in analogous experimental systems to mitigate loading variability and ensure quantification accuracy [34,35].

### 4.9. Statistical Analysis

Data analysis was conducted using IBM SPSS 28.0.1. Continuous data are expressed as mean ± standard deviation (SD). For multiple group comparisons, initial analysis was performed using one-way analysis of variance (ANOVA) followed by post hoc Fisher’s least significant difference (LSD) test. Behavioral changes were analyzed using Repeated-Measures two-way ANOVA. Pearson correlation was used to determine the correlation between the intensity of TRPV1 expression (western blotting) and behavioral performance. Statistical significance was accepted at the level of *p* < 0.05 with asterisks and sharp symbols in figures denoting *p* values as follows: * *p* < 0.05, ** *p* < 0.01, *** *p* < 0.001 and # *p* < 0.05, ## *p* < 0.01, ### *p* < 0.001.

## 5. Conclusions

In conclusion, this study successfully developed an experimental tooth movement (ETM) model using a 50 g force and investigated the expression of TRPV1 in the CeA. The findings indicate that TRPV1 expression in the CeA was significantly upregulated following force application, implying that TRPV1 may play a role in mediating tooth movement-induced pain in rats. These results establish a foundation for future studies to clarify the functional role of TRPV1 in this pathway.

## Data Availability

The data that support the findings of this study are available on request from the corresponding author.
